# Phase separation as an emerging regulatory framework in antibody class switching and genome stability

**DOI:** 10.3389/fimmu.2026.1817853

**Published:** 2026-04-15

**Authors:** Ahmed M. Refaat, Hidetaka Shimizu, Takaaki Yasuhara

**Affiliations:** 1 Laboratory of Genome Stress Response, Radiation Biology Center, Graduate School of Biostudies, Kyoto University, Kyoto, Japan; 2 Zoology Department, Faculty of Science, Minia University, El-Minia, Egypt

**Keywords:** activation induced cytidine deaminase (AID), biomolecular condensates, class switch DNA recombination (CSR), DNA repair, genomic instability, liquid liquid phase separation (LLPS)

## Abstract

The spatial and temporal organization of nuclear processes is increasingly interpreted through principles associated with liquid–liquid phase separation (LLPS), whereby multivalent interactions among proteins and nucleic acids generate dynamic, membraneless assemblies. In DNA repair, such assemblies have been proposed to coordinate damage sensing, signaling, and repair pathway choice; however, their causal contribution in physiological immune contexts remains under active investigation. Antibody class switch recombination (CSR) provides a stringent immunological model in which to examine these concepts, as activated B lymphocytes must efficiently rejoin programmed DNA double-strand breaks (DSBs) across long genomic distances while suppressing aberrant chromosomal rearrangements. Emerging evidence indicates that CSR involves dynamic RNA–protein assemblies enriched for 53BP1, heterogeneous nuclear ribonucleoproteins such as HNRNPU, and transcription-associated RNA scaffolds, with properties consistent with biomolecular condensation. These assemblies are proposed to function as a CSR-specific regulatory hub—or “switchosome”—that concentrates non-homologous end joining factors, enforces repair pathway choice, and integrates transcription, RNA structure, and chromatin architecture at immunoglobulin heavy-chain (*IgH*) switch regions. Rather than treating LLPS as universally established, this review critically evaluates experimental evidence supporting condensate-like behavior in CSR-associated repair compartments, distinguishing demonstrated mechanisms from LLPS-consistent or speculative models. We further discuss how disruption of condensate dynamics—either through impaired assembly or pathological stabilization—can compromise repair fidelity, contributing to immunodeficiency and B cell lymphomagenesis. By positioning CSR as a paradigm for studying higher-order nuclear organization during programmed genome rearrangements, this review highlights how condensate-based regulation may contribute to immune diversification and genome stability.

## 1 Introduction

The organization of the eukaryotic nucleus, long understood in terms of membrane-bound compartments and chromatin architecture, is increasingly interpreted through principles of biomolecular condensation. In this context, condensate refers to the thermodynamic process by which multivalent interactions among macromolecules drive the demixing of a homogeneous solution into concentrated molecular assemblies. The resulting condensates are membraneless compartments enriched in specific proteins and nucleic acids that locally concentrate biochemical reactions. Many such assemblies display liquid-like physical properties—including spherical morphology, fusion behavior, and rapid molecular exchange—and are therefore often described as droplet-like structures. While many biomolecular condensates are described as liquid droplets formed through liquid–liquid phase separation (LLPS), we use the broader term bio molecular condensate to denote membraneless assemblies that concentrate macromolecules. Importantly, such assemblies can arise not only through LLPS but also through related mechanisms, including polymer-mediated clustering or gel-like network formation, processes that often involve intrinsically disordered proteins (IDPs) and nucleic acids (1–3). Such condensates underlie the formation of numerous nuclear and cytoplasmic compartments, including nucleoli, stress granules, and promyelocytic leukemia (PML) bodies, where they function as hubs that concentrate specific macromolecules to facilitate or regulate biochemical processes (4, 5). This principle provides a conceptual framework for nuclear compartmentalization, most prominently in transcriptional regulation, where master regulators like the Mediator complex and bromodomain-containing protein 4 (BRD4) form condensates at super-enhancers to drive robust gene expression programs (6). LLPS has also been implicated in higher-order genome organization, including the formation of repressive heterochromatin domains mediated by HP1α and the segregation of chromatin into distinct physical and functional compartments (7).

The capacity of LLPS to create precise biochemical environments with high spatiotemporal control may also contribute to genome maintenance. Emerging evidence suggests that this physicochemical principle is co-opted to orchestrate the DNA damage response (DDR), where it serves as a critical regulator by forming specialized repair hubs. Early work showed that the poly(ADP-ribose) (PAR) polymer synthesized by PARP1 at DNA lesions can act as a seeding scaffold for the phase separation of DDR factors such as FUS and TAF15 (8). Furthermore, the central DDR organizer 53BP1 itself undergoes LLPS, forming condensates that scaffold downstream repair effectors and amplify the damage signal (9). These LLPS-associated assemblies thus create dedicated compartments that concentrate repair machinery, bias pathway choice, and support DNA repair fidelity.

The functional implications of LLPS are particularly evident in antibody class switch recombination (CSR), a physiological paradigm of programmed genomic instability. During CSR, activated B lymphocytes intentionally generate DNA double-strand breaks (DSBs) within the immunoglobulin heavy-chain (*IgH*) locus to diversify antibody effector functions. This process is initiated by activation-induced cytidine deaminase (AID), which targets actively transcribed switch (S) regions, and is primarily resolved through non-homologous end joining (NHEJ) (10–12). Despite extensive biochemical and genetic analyses, how AID is selectively recruited to switch regions within *IgH* locus remains incompletely understood. Canonical models based on transcriptional activity, R-loop formation, or sequence motifs alone cannot fully account for the robustness and specificity of AID targeting CSR, particularly given that transcription and R-loop–forming sequences are widespread throughout the genome. This gap highlights the need for a mechanistic framework that explains how switch regions generate a privileged local environment for AID action. The central challenge of CSR lies in efficiently synapsing and joining distant DSBs within the *IgH* locus while suppressing aberrant repair outcomes, such as chromosomal translocations, that drive B cell lymphomagenesis (13). Classical models based solely on stoichiometric recruitment of repair factors fail to fully explain how such precise spatiotemporal coordination is achieved over long genomic distances and in the presence of potentially destabilizing structures such as R-loops.

Emerging evidence suggests that LLPS may provide a physical mechanism capable of resolving these constraints. Where appropriate, we distinguish mechanisms directly demonstrated in B cells during CSR from organizing principles inferred from other cellular systems that remain consistent with LLPS-based models. Several CSR regulators, including 53BP1 and the RNA-binding protein HNRNPU, have been reported to assemble into dynamic repair compartments proposed to function as a molecular “switchosome” that promotes repair fidelity (9, 14, 15). These assemblies are proposed to concentrate NHEJ components, stabilize *IgH* chromatin topology, and suppress error-prone repair pathways such as alternative end joining (A-EJ), also referred to as microhomology-mediated end joining (MMEJ), by compartmentalizing the reaction environment. Importantly, disruption of condensate dynamics—for example, through aberrant AID condensation observed in Hyper-IgM syndrome—directly links defective phase separation to impaired CSR and genomic instability (16, 17).

This review proposes that LLPS represents a regulatory layer in CSR, organizing repair factors into transient, dynamic condensates that function as dedicated repair compartments and support genome integrity during programmed DNA rearrangements.

## 2 The functional architecture of DNA repair condensates

### 2.1 Amplification and threshold sensing: building a robust damage signal

**Figure 1:**
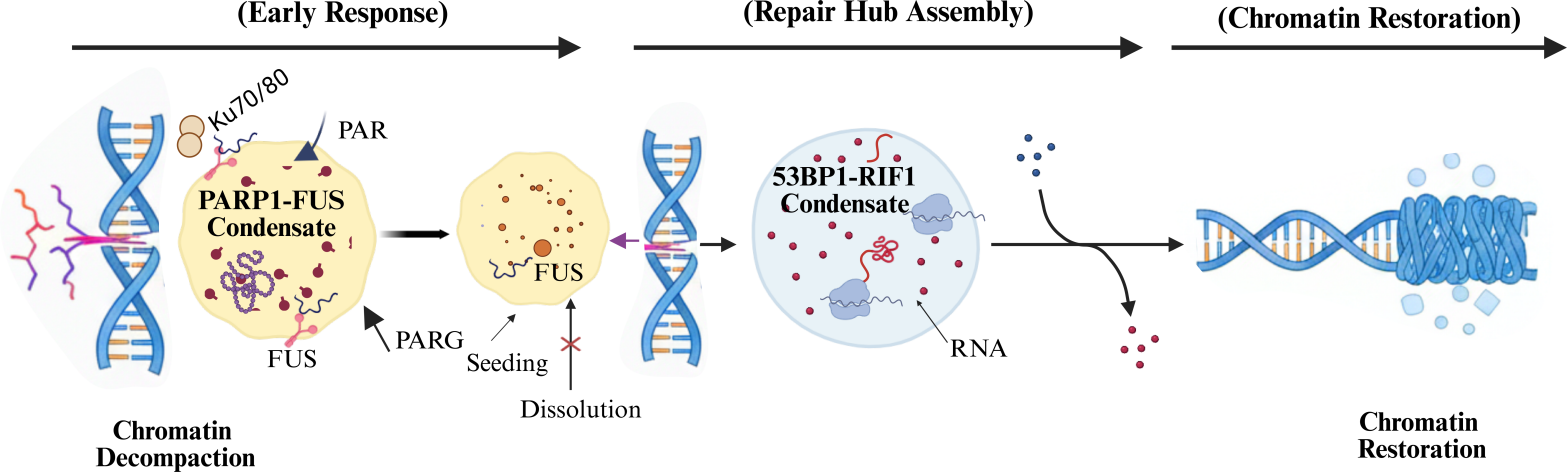
Stepwise assembly and resolution of phase-separated repair condensates during the DNA damage response. Schematic representation of the temporal organization of liquid-liquid phase separation (LLPS)-associated assemblies during the DNA double- strand break (DSB) repair response. In the early phase, PARP1 activation generates poly(ADP-ribose) (PAR) chains that promote chromatin relaxation and nudeation of transient, liquid-like protein assemblies enriched in intrinsically disordered factors such as FUS, facilitating rapid recruitment of early DNA damage response components. During the intermediate phase, RNA polymerase II-dependent synthesis of damage- induced long noncoding RNAs (dilncRNAs) supports the formation of RNA-dependent assemblies containing 53BP1 and RIF1, spatially organizing core repair machineries at break sites. In the late phase, completion of repair is accompanied by PARG-mediated PAR removal, condensate dissolution, and chromatin re-compaction, restoring nudear architecture. Arrows indicate temporal progression, and distinct chromatin and condensate states are indicated by color coding.

CSR represents a uniquely programmed form of genome instability in activated B cells, requiring the rapid amplification of stochastic DNA lesions while maintaining strict spatial confinement to immunoglobulin loci. A central challenge in this process is converting sparse, transcription-coupled DNA damage events into a robust and locus-restricted repair signal that ensures productive recombination while suppressing off-target rearrangements. Increasing evidence suggests that spatial organization principles commonly associated with LLPS contribute to this amplification and threshold-sensing behavior, with individual assemblies likely spanning a continuum of material states rather than representing uniform phase-separated condensates (14, 18). The universal response to DSBs is initiated within seconds by binding the Ku70/80 heterodimer to DNA ends, followed closely by activation of PARP1 and the synthesis of PAR. PARylation promotes local chromatin relaxation and generates a transient molecular scaffold that enhances recruitment of early DDR factors (19–21). PAR chains also recruit IDPs, including members of the FET family (FUS, EWS, and TAF15), as demonstrated in several non-B-cell DNA damage models, together with additional DNA- and RNA-binding factors. These interactions promote the formation of dynamic, highly concentrated protein assemblies at sites of damage, facilitating the local enrichment of DDR components such as MRE11, MDC1, and 53BP1 and accelerating early steps of DSB processing (8, 22, 23). This PAR-dependent condensation represents one of the earliest organizational steps after DNA damage, linking metabolic signaling to the compartmentalization of repair. The transient nature of these initial condensates is balanced by the counteracting enzyme PARG (poly ADP-ribose glycohydrolase), whose de-PARylation activity dissolves PAR scaffolds to reset the repair environment (23) (**Figure 1**).

**Figure 2:**
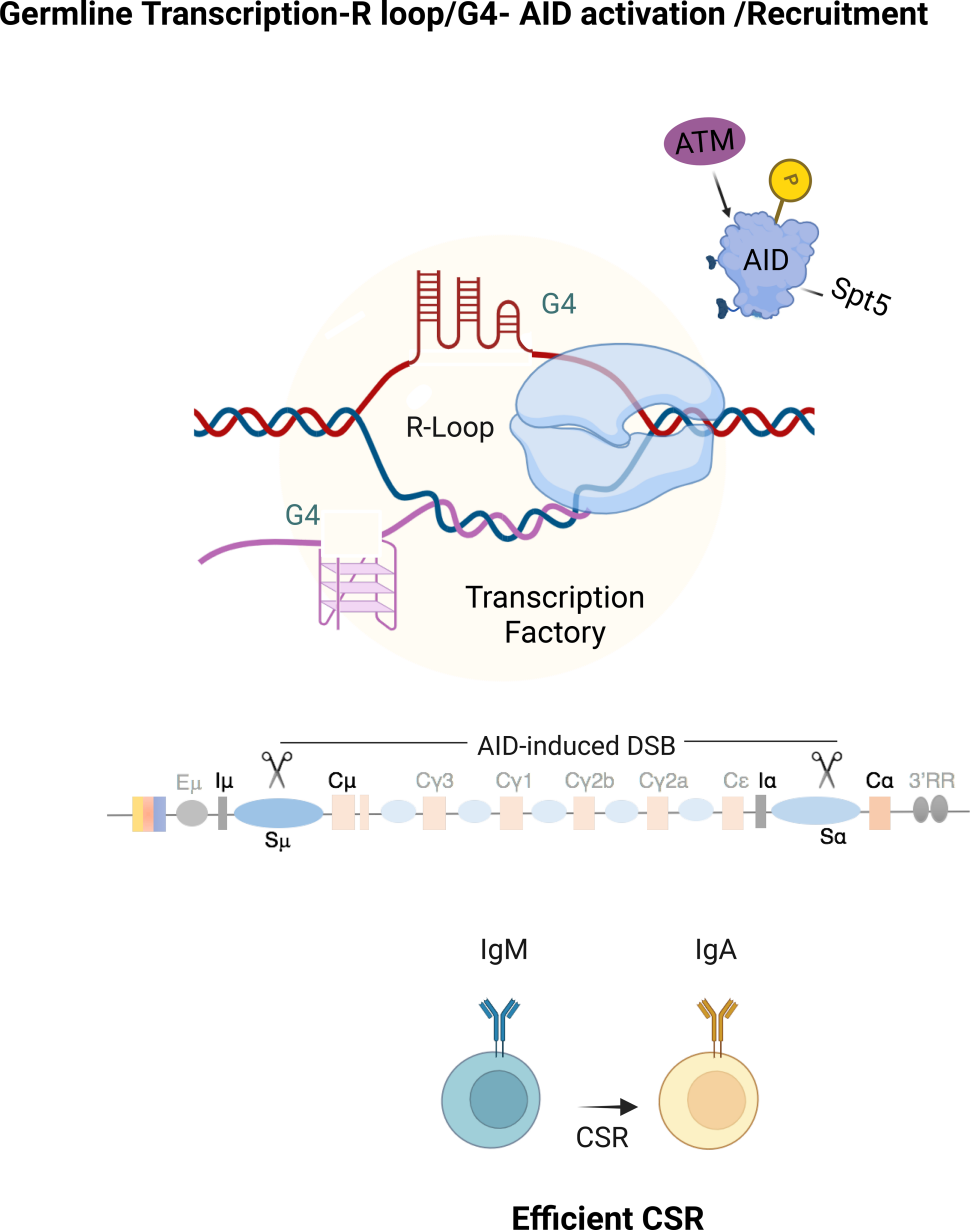
R-loop and g-quadruplex structures coordinate ATM-8pt5-AID activation during CSR. Proposed model illustrating how transcription-coupled nucleic acid structures facilitate activation of AID during class CSR. Intense transcription over immunoglobulin heavy-chain (*IgH*) switch regions generates a local transcription hub enriched in RNA polymerase II, transcription cofactors, and nascent germline transcripts. Within this environment, R-loops and G-quadruplex (G4) structures form over switch regions, creating a structural platform that promotes recruitment of phosphorylated AID together with regulatory factors, including ATM and Spt5. By analogy to RNA-dependent repair assemblies described for other DNA repair contexts, these structures may exhibit sensitivity to perturbation of multivalent interactions. Arrows indicate progression toward productive CSR and isotype switching.

Within CSR, this early spatial concentration of repair and signaling factors provides a physical framework for biochemical signal amplification. Following AID recruitment and cytidine deamination at switch (S) regions, ATM activation leads to phosphorylation of AID at serine 38, promoting interaction with the endonuclease APE1 and enhancing conversion of lesions into DSBs (24) (**Figure 2**). This feed-forward loop—where initial breaks stimulate further break formation—ensures that damage density surpasses a functional threshold required for productive long-range synapsis. Spatial confinement of this amplification cycle is critical, as excessive or mislocalized break formation would increase the risk of oncogenic translocations.

As PAR-dependent assemblies resolve, downstream repair organization is shaped by larger and more stable 53BP1-enriched nuclear compartments. These structures are supported, in part, by RNA species transcribed in the vicinity of DSBs and exhibit dynamic behaviors consistent with biomolecular condensation, including fusion, fission, and rapid molecular exchange (9, 14) (**Figure 1**). These assemblies exhibit behaviors consistent with LLPS, independent of upstream signals such as γH2AX and MDC1, serving as structural scaffolds for downstream effectors, stabilizing p53, and enhancing its transcriptional responses, linking LLPS to cell fate after genotoxic stress (9, 18). While these behaviors are consistent with LLPS, alternative models involving polymer–gel or scaffold-based assemblies cannot yet be fully excluded.

CSR imposes additional specialization on these organizing principles through its tight coupling to transcription. Germline transcription through S regions generates R-loops and G-quadruplex (G4) structures that expose single-stranded DNA substrates for AID activity (25). Efficient germline transcription, which generates the RNA scaffolds for AID recruitment, is controlled by factors that regulate RNA polymerase II pause-release and elongation, including Spt5, PAF1, and ELOF1 (26–28). Efficient CSR initiation depends on AID targeting to chromatin enriched for R-loops and G4s at S regions (29–31). Because transcription itself can be spatially concentrated through dynamic transcriptional assemblies, local enhancement of transcriptional activity may indirectly increase R-loop formation and stability, reinforcing locus specificity during CSR initiation (32, 33). Given the high frequency and spatial separation of AID-induced lesions during CSR, fidelity cannot be ensured by factor recruitment alone but instead requires confinement of recombination events within a restricted nuclear reaction space. By promoting high local transcriptional activity at S regions, phase-separated transcriptional hubs may increase R-loop abundance and stability, thereby facilitating AID access and reinforcing the spatial specificity of the initial recombination signal. Consistent with this view, AID interacts with the transcription elongation factor Spt5 and localizes preferentially to paused RNA polymerase II, directly linking transcriptional stress to cytidine deamination (26) (**Figure 2**).

**Figure 3:**
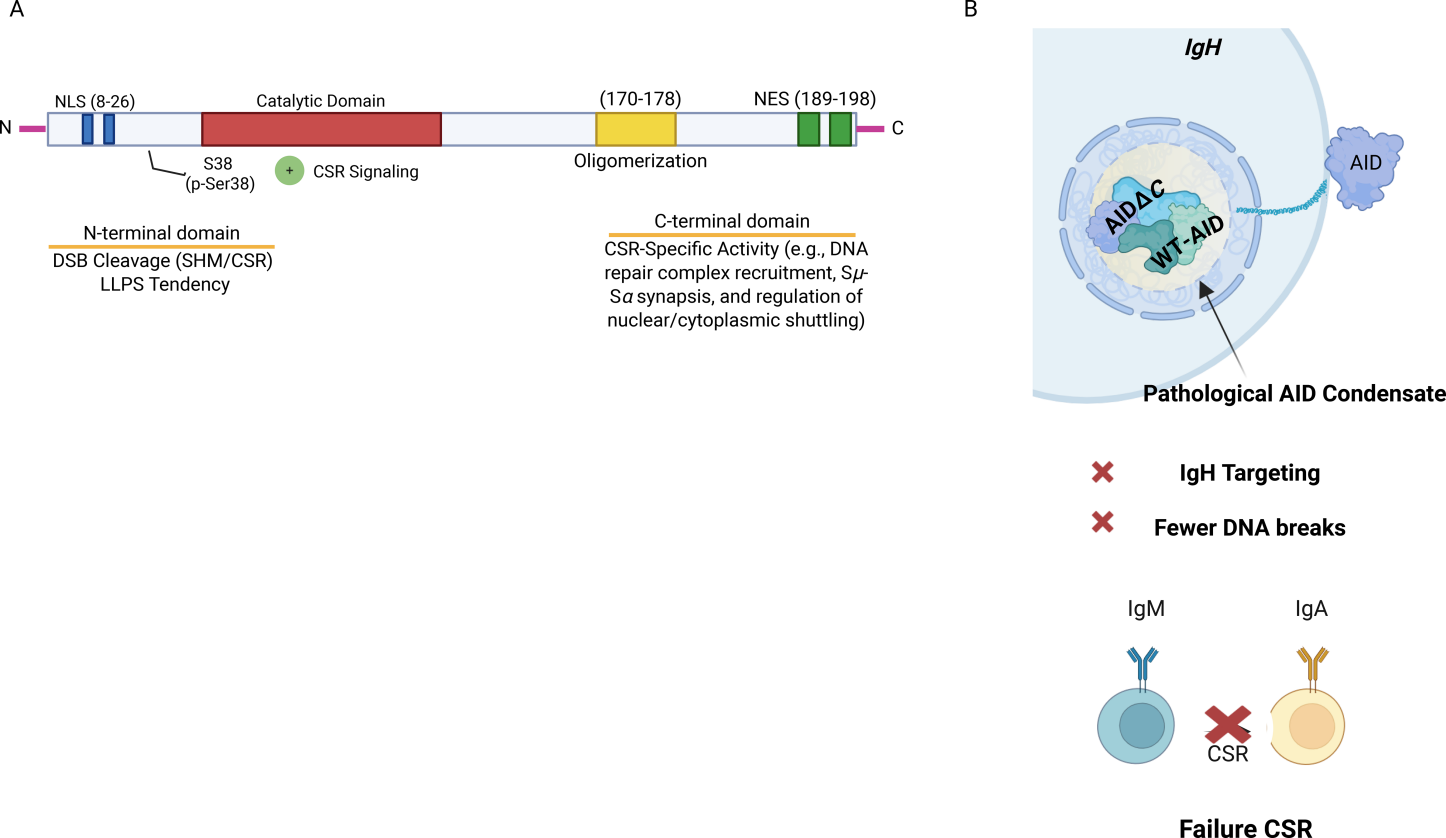
R-Loop/G4-enriched condensate assemblies regulate AID function during CSR. **(A)** Domain organization of AID, highlighting the catalytic domain (CD), nuclear localization signal (NLS), nuclear export signal (NES), and C-terminal regulatory region. Conserved motifs essential for AID trafficking and enzymatic activity are indicated. **(B)** Model illustrating how C-terminal truncations of AID (ΔC-AID) lead to aberrant nuclear accumulation and formation of dysfunctional assemblies that sequester wild-type AID. These aberrant assemblies impair proper targeting of AID to *IgH* switch regions, providing a mechanistic explanation for the dominant-negative behavior observed in Hyper-IgM syndrome type 2.

Although AID activity is amplified by phosphorylation signaling, its access to the *IgH* locus is tightly regulated by subcellular localization, a process susceptible to LLPS dynamics: wild-type AID (AID^WT) is exported out of the nucleus via its C-terminal nuclear export signal (NES), predominantly residing in the cytoplasm, whereas C-terminal deletion mutants (AIDΔC, such as R190X or V186X) accumulate constitutively in the nucleus yet fail to support efficient CSR (16). These mutants form nuclear puncta with dynamic properties characteristic of biomolecular condensation, including rapid fluorescence recovery after photobleaching and sensitivity to 1, 6-hexanediol. Condensation propensity arises from intrinsic sequence features within the AID N-terminus, including intrinsically disordered regions and a surface-exposed arginine-rich patch (16). Functionally, these aberrant assemblies fail to colocalize with the endogenous *IgH* locus and induce fewer programmed breaks. Moreover, in cells co-expressing wild-type and mutant AID, AIDΔC sequesters functional AID into nucleolar-associated compartments marked by fibrillarin, exerting a dominant-negative effect that suppresses CSR (16) (**Figure 3**). Together, these observations support a model in which CSR relies on finely tuned spatial concentration mechanisms to amplify DNA damage signals while enforcing locus specificity. Perturbation of these organizing principles—whether through insufficient assembly formation or through hyper-stable, mislocalized condensates—selectively compromises antibody diversification and increases susceptibility to aberrant chromosomal rearrangements and lymphomagenesis.

### 2.2 RNA-mediated condensates: damage-induced lncRNAs and RNA–protein networks

RNA molecules serve as central regulators of RNA-dependent condensation and LLPS-like organization within DDR, functioning as both structural scaffolds and dynamic modulators of condensate formation. These RNA-dependent mechanisms are particularly specialized in CSR, where they coordinate the initiation, spatial confinement, and faithful resolution of programmed DNA breaks at the *IgH* locus. Nascent RNAs generated near DSBs engage in multivalent RNA–protein and protein–protein interactions, thereby nucleating or reinforcing localized repair compartments (34). These RNA-dependent assemblies provide a physical framework for concentrating repair factors and coordinating signaling, consistent with principles commonly associated with biomolecular condensation.

**Figure 4:**
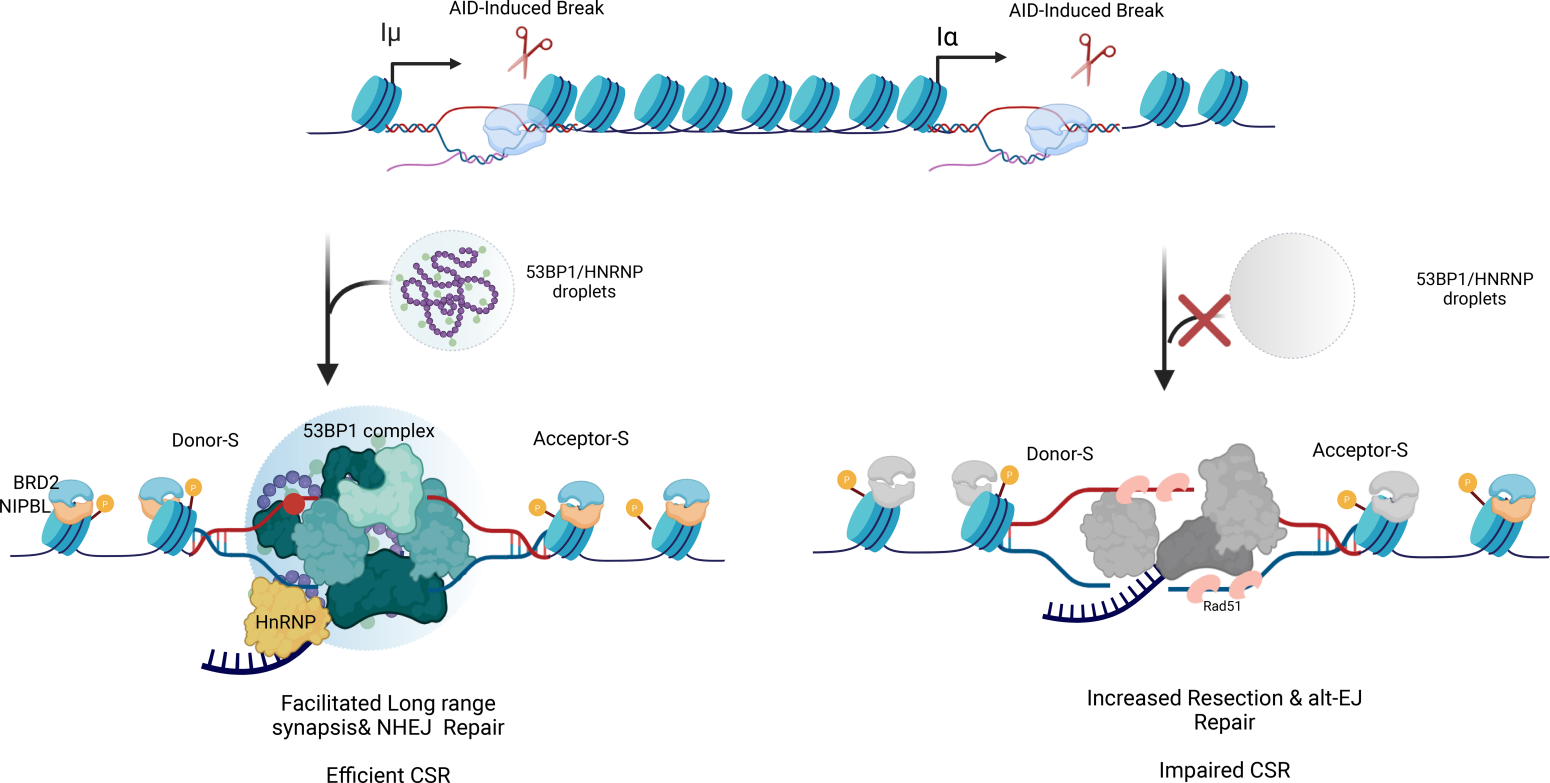
LLPS-dependent end protection by the 53BP1 complex enforces canonical NHEJ during CSR. ntribute to DNA end protection at *IgH* DSBs. A cohesive repair assembly containing 53BP1, RIF1, and Shieldin forms at break sites, stabilizing Ku80 binding and preventing nucleolytic resection, thereby favoring canonical non-homologous end joining (C-NHEJ). hnRNPU further reinforces end protection through RNA- and G4-RNA -dependent interactions, analogous to its reported functions at telomeric regions. Disruption or failure of condensate formation leads to loss of end protection, increased ssDNA generation, engagement of resection-associated factors such as Rad51, and a shift toward alternative end joining (alt-EJ), resulting in aberrant CSR junctions.

In CSR, this general RNA-driven organizational logic is adapted through a network of locus-specific transcripts. The process is initiated by GLTs transcribed from S regions, which form R-loops that expose single-stranded DNA substrates for AID targeting (35). Additionally, the G4 structures adopted by these transcripts function as sequence-encoded guide RNAs that facilitate AID recruitment to S-region chromatin (11, 36). Zheng and colleagues demonstrated that AID binds directly to switch region RNA through G-quadruplex structures, and that intronic RNA can act in trans to target AID to DNA (30). This interaction was structurally elucidated by Qiao et al. (36), who solved the AID crystal structure and showed that G-quadruplex substrates induce cooperative AID oligomerization, providing a biophysical basis for its high-affinity binding to structured nucleic acids. While G-quadruplexes formed by G-rich switch regions provide high-affinity binding sites for AID, they are not absolutely required for CSR, as AT-rich sequences from Xenopus can substitute and support recombination in mouse B cells (37). This suggests that high local transcriptional density and the generation of multivalent RNA platforms, rather than a specific secondary structure, may represent the core requirement for AID recruitment. Beyond their targeting role, G4-containing GLTs act as potent multivalent scaffolds capable of promoting biomolecular condensation (38). Their repetitive, G-rich architecture provides multivalent interaction surfaces for IDR- and RGG-domain–containing proteins such as FMRP and heterogeneous nuclear ribonucleoproteins (hnRNPs) (39), enhancing the nucleation and stability of CSR-specific RNA–protein assemblies. Recent work has reframed germline switch transcripts not merely as byproducts of transcription or passive substrates for R-loop formation, but as active molecular components capable of shaping the local nuclear environment (40). Switch region RNAs are unusually abundant, sequence-repetitive, and enriched in secondary structure–forming motifs, properties that distinguish them from most intronic transcripts. These features suggest that switch RNAs may contribute to spatial organization at the *IgH* locus by serving as scaffolding elements for protein recruitment and retention. RNA-dependent interactions directly contribute to the organization of repair machinery. G4-rich GLTs promote HNRNPU-dependent assemblies through specific RGG-domain interactions, generating concentrated repair environments enriched in core NHEJ components, including Ku80, DNA-PKcs, 53BP1, and the Shieldin complex (15) (**Figure 4**). HNRNPU may further stabilize long-range synapsis by recruiting additional scaffolding non-coding RNAs, such as LINP1, which bridges Ku80 and DNA-PKcs (15). Enhancer-derived RNAs provide an additional regulatory layer: eRNAs transcribed from the *IgH* 3′ regulatory region (3′RR) interact with the hnRNPL–CstF64 complex to stabilize 53BP1 and Ku80 recruitment at both switch and enhancer regions (41). Although these assemblies have not been formally demonstrated to represent canonical phase-separated droplets, their enrichment in IDR-containing proteins and their dynamic behavior suggest a condensation-like organizational mechanism. Direct experimental demonstration of LLPS during CSR, however, remains limited. These RNA-dependent structures may therefore provide a framework that spatially couples transcriptional activity to NHEJ factor recruitment. Together, these observations position RNA not merely as a targeting signal but also as a structural determinant of CSR repair microenvironments.

The fidelity of this RNA-driven microenvironment is maintained by multiple quality-control pathways. The RNA exosome resolves excessive or persistent R-loops, while m^6^A modification of GLTs enables regulated transcript turnover via YTHDF reader proteins, preventing unscheduled or ectopic recombination events (42). Furthermore, RNA Polymerase II synthesizes dilncRNAs that seed 53BP1 condensates—key LLPS structures for efficient NHEJ (8). Through their IDRs and RGG motifs, 53BP1 oligomerizes in an RNA-dependent manner to recruit RIF1, REV7, and Shieldin (9), thereby linking transcription, RNA structure, and repair enzyme localization within an LLPS-driven microenvironment (**Figure 4**).

This sophisticated system is vulnerable to dysregulation, illustrating the critical importance of precise RNA–protein coordination. Under conditions of transcriptional stress, paraspeckle-associated factors can aberrantly assemble on ribosomal RNA rather than their canonical scaffold NEAT1 (43), illustrating how misdirected RNA binding can rewire nuclear organization. Similarly, PARP1-mediated FUS condensation coordinates RNA scaffolds with early DDR signaling, demonstrating a multilayered network of RNA-dependent assembly events (34, 44).

Related RNA-driven organizational principles are observed at telomeres, where repetitive sequences transcribe the telomere repeat-containing RNAs that interact directly with shelterin complex subunits TRF1 and TRF2 (1, 45), partitioning into telomeric droplets to preserve condensate stability and chromosome-end protection (45). Collectively, this hierarchy of RNA species—from universal DDR-associated dilncRNAs to locus-specific guides and stabilizers such as G4-GLTs and 3′RR eRNAs—establishes RNA as a central architect of the CSR repair environment. By conferring specificity, structural integrity, and regulatory checkpoints, RNA-driven assemblies enable the confinement of programmed DNA recombination to the *IgH* locus while minimizing the risk of pathological chromosomal rearrangements.

### 2.3 Repair pathway choice: condensation-based environments influencing the NHEJ–HR balance

Following the initial PARP1- and RNA-driven condensate formation, the cell must determine which DSB repair route to pursue—NHEJ or homologous recombination (HR) —in both genome maintenance and CSR. This decision is not dictated solely by enzymatic activities but is strongly influenced by spatial organization within the nucleus. Increasing evidence indicates that condensation-based microenvironments—some exhibiting liquid-like properties—play a central role in biasing repair outcomes by selectively concentrating or excluding pathway-specific factors.

Building on early FUS-initiated 53BP1 condensates, the master NHEJ mediator 53BP1 serves as a scaffold for RIF1 and the Shieldin complex to create a barrier against DNA end resection—a function particularly critical when sister chromatids are unavailable (9, 14, 46). In activated B cells, genetic disruption of Shieldin components, including REV7, results in increased DNA end resection at immunoglobulin switch regions, elevated microhomology usage, and impaired CSR efficiency, demonstrating that pathway choice at the *IgH* locus is actively enforced toward NHEJ rather than emerging passively from enzymatic competition (47, 48). Conversely, conditions that permit inappropriate accumulation of homologous recombination factors at switch regions—such as loss of 53BP1 pathway components or perturbation of chromatin organization—correlate with defective CSR and increased chromosomal aberrations, underscoring the necessity for spatial exclusion of HR during antibody diversification (46, 48, 49).

These 53BP1-enriched assemblies display dynamic behaviors consistent with biomolecular condensation and create a resection-refractory environment that favors direct ligation. Importantly, these structures are not static. During S/G2 phase, when HR is preferred, BRCA1 antagonizes 53BP1 accumulation at damage sites, permitting DNA end resection (50). Rather than simple dissolution, this transition appears to involve spatial reorganization, displacement, or encapsulation of 53BP1 assemblies by BRCA1-containing complexes (51), highlighting how repair pathway switching can be achieved through remodeling of local repair environments.

**Figure 5:**
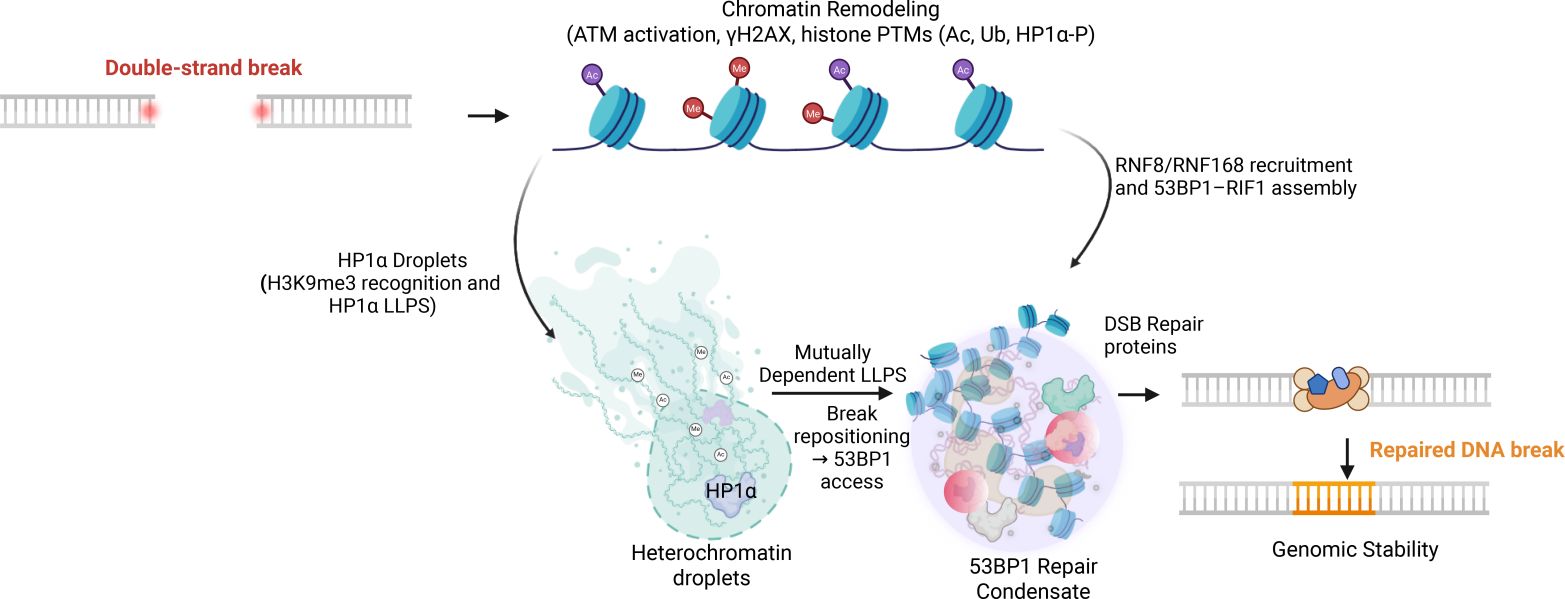
LLPS-mediated coordination of chromatin remodeling and DNA repair condensates. DSB triggers ATM activation, γH2AX spreading, and rapid chromatin remodeling, including histone acetylation, ubiquitination, and HP1αphosphorylation. These early modifications facilitate formation of HP1α-enriched assemblies through recognition of H3K9me3 and LLPS-associated multivalent interactions, establishing a heterochromatin environment conducive to break repositioning. Concurrently, acetylated and ubiquitinated chromatin promotes the assembly of phase-separated 53BP1 -RIF1 repair condensates. Cross-talk between HP1αdroplets and 53BP1 condensates ensures mutually dependent LLPS, stabilizing repair hubs and integrating chromatin state with repair pathway choice to enable efficient DSB repair.

Related principles operate in specialized chromatin contexts. In heterochromatic regions, Nup98 forms protective, condensation-dependent assemblies at DSBs that actively exclude RAD51, thereby suppressing inappropriate HR and maintaining genome stability (52). These examples illustrate how repair pathway choice is regulated through reversible, spatially defined compartmentalization, providing a tunable layer of control that complements canonical signaling pathways (**Figure 5**).

In CSR, where AID-induced DSBs require productive C-NHEJ-mediated joining, pathway enforcement is achieved through specialized RNA-protein condensates. HNRNPU-dependent assemblies create NHEJ-permissive environments at switch regions, with disruption of these condensates leading to increased microhomology usage and reduced CSR efficiency (15). This pathway bias is further reinforced by R-loop regulation, as persistent RNA–DNA hybrids can interfere with the 53BP1–Shieldin protective complex, promoting resection-prone substrates and repair outcomes associated with A-EJ (15, 42, 53). Additional condensation-based regulation involves chromatin-associated factors such as BRD2 and NIPBL, whose loss destabilizes NHEJ-supportive assemblies, promotes inappropriate RAD51 recruitment, and skews repair toward HR/A-EJ (54) (**Figure 4**).

Conversely, HR-associated factors also exploit condensation-like behaviors to coordinate their sequential activities. In transcriptionally active chromatin, R-loops can promote HR by facilitating end resection and homology-directed repair under appropriate cell-cycle conditions (55, 56). In yeast, Rad52 exhibits droplet-like behavior *in vitro*, providing an evolutionary model for how multivalent interactions can facilitate the dynamic assembly required for strand invasion and homology search (57). Similarly, RPA32, a subunit of the RPA complex that coats ssDNA, exhibits LLPS behavior driven by ssDNA and phosphorylation (57). The subsequent replacement of RPA by RAD51, a critical step for HR initiation, also needs efficient spatial organization (58). These observations suggest that repair pathway choice emerges from the dynamic interplay between distinct condensation-based environments: more fluid assemblies facilitate factor recruitment and homology search, whereas more stabilized structures protect DNA intermediates and enforce pathway commitment. This equilibrium is further shaped by nuclear architecture, as DSB relocation to nuclear pores or the nuclear periphery can suppress HR and promote NHEJ or microhomology-mediated end joining (44).

Collectively, these findings support a model in which condensation-based nuclear organization provides a unifying framework for repair pathway choice. By modulating local composition, dynamics, and accessibility, these assemblies ensure repair fidelity through spatial control, complementing enzymatic regulation and enabling context-dependent tuning of genome maintenance processes.

### 2.4 Integration with chromatin dynamics: condensate-mediated chromatin remodeling at damage sites

Emerging evidence suggests that principles associated with LLPS contribute to the spatial organization of chromatin across multiple scales, ranging from local nucleosome arrays to higher-order topological domains. Chromatin has been proposed to exhibit condensation behavior driven by multivalent interactions among histone tails, DNA, and chromatin-associated proteins, contributing to the segregation of euchromatic (transcriptionally active) and heterochromatic (repressive) regions (1, 44). This property arises from the intrinsically disordered histone tails and their post-translational modifications (PTMs). Histone acetylation disperses chromatin condensates and promotes transcriptional activity, whereas hypoacetylated histones and the linker histone H1 favor chromatin compaction and phase separation (59). The maintenance of heterochromatin integrity relies heavily on multivalent protein networks with phase separation properties. Core heterochromatin protein HP1α undergoes LLPS with H3K9me3-marked chromatin to form stable pericentromeric foci, with its phase behavior regulated by DNA binding and N-terminal phosphorylation that enhances multivalency (60, 61). This architectural role extends to the DSB repair regulator 53BP1, which engages in mutually dependent LLPS with HP1α to maintain heterochromatic integrity and repress repetitive elements—a function distinct from its canonical repair role (62). Additional structural reinforcement is provided by the nuclear matrix protein SAFB, which forms assemblies with HP1α and repetitive element–derived RNAs, stabilizing heterochromatin organization and maintaining chromatin insulation (63) (**Figure 5**).

At DNA damage sites, condensate-associated mechanisms contribute to dynamic chromatin remodeling that facilitates repair. In heterochromatic regions, the liquid-like properties of HP1α-containing assemblies enable damaged loci to relocate outside compact domains, thereby permitting efficient repair while preserving overall heterochromatin integrity (34, 44). This movement underscores the dynamic interplay between heterochromatin condensates and DNA repair condensates. In euchromatic regions, nucleosome arrays themselves can form dynamic assemblies whose properties are modulated by histone acetylation and bromodomain-containing readers. BRD4, through its bromodomains and intrinsically disordered regions, forms immiscible assemblies on acetylated chromatin, organizing transcriptionally active domains (64). Through this mechanism, BRD4 not only sustains active transcriptional hubs but also promotes NHEJ by scaffolding 53BP1 and other repair factors at DSBs (65). Histone acetyltransferases CBP/p300 further amplify this process by acetylating chromatin flanking DNA breaks, promoting SWI/SNF recruitment and nucleosome remodeling to enhance repair factor accessibility (66).

Beyond local chromatin remodeling, condensate-associated assemblies are proposed to influence higher-order chromatin topology. 53BP1/RIF1 assemblies stabilize γH2AX-marked chromatin loops and confine signaling within topologically associating domain (TAD) boundaries, preserving local three-dimensional structure (67). These condensates exert physical forces that cluster genomic regions and counteract decompaction, with 53BP1 loss leading to topological distortion and disrupted domain integrity (44, 67). In parallel, DSBs trigger the reorganization of cohesin-mediated loop extrusion, allowing ATM kinase activity to spread across flanking chromatin while remaining constrained within TADs (68). Condensate-associated forces may contribute to this process by clustering genomic regions and coordinating biochemical signaling with large-scale chromatin architecture.

**Figure 6:**
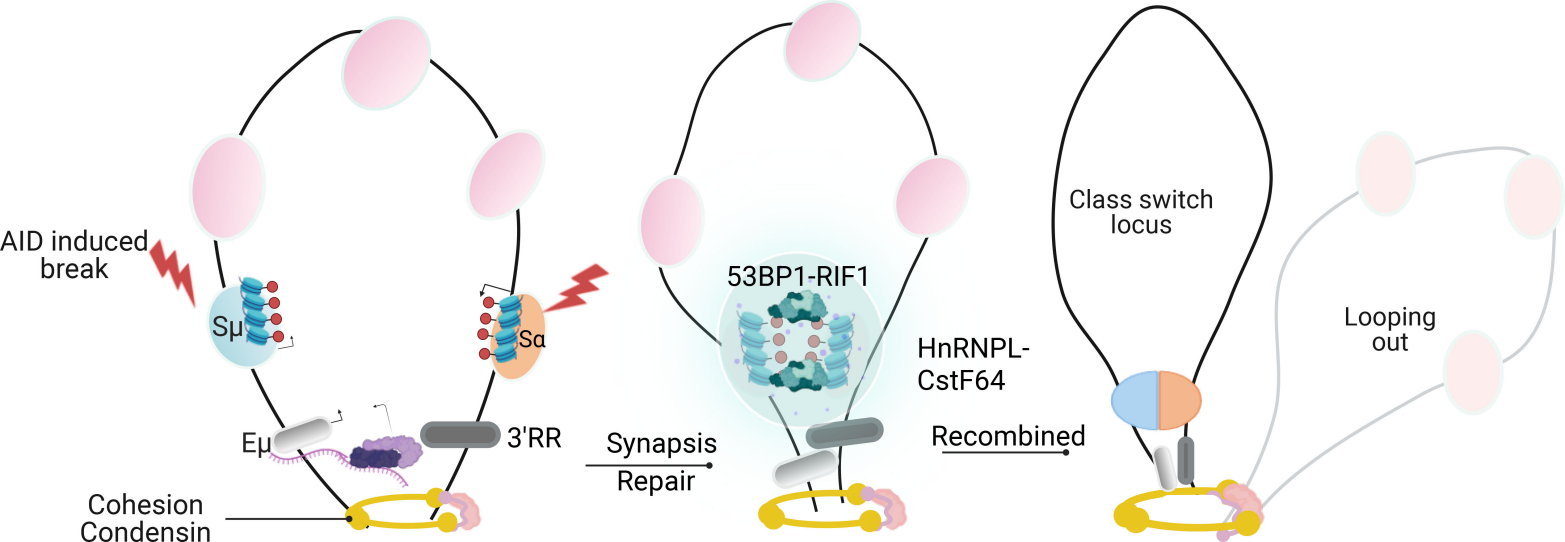
Loop Extrusion and RNA-Dependent Assemblies Organize Switch-Region Synapsis During CSR. Model of how higher-order *IgH* locus architecture and RNA-dependent assemblies coordinate switch-region synapsis. Cohesin-mediated loop extrusion juxtaposes donor Sμand acceptor S regions, bringing the 5′ Eμenhancer into proximity with the 3′ regulatory region (3′ RR). Enhancer-derived RNAs (eRNAs) from the 3′ RR interact with hnRNPL and CstF64, forming an RNA-dependent scaffold that promotes recruitment of 53BP1 and Ku80 to synapsed switch regions. Although direct phase separation has not been formally demonstrated in this context, enrichment of intrinsically disordered proteins suggests a condensate-like mechanism. Stabilization of synapsis by 53BP1 -RIF1 assemblies constrains DNA damage signaling and promotes efficient C-NHEJ, integrating *IgH* topology with repair factor organization during CSR.

In CSR, these organizing principles are deployed to reconfigure the three-dimensional architecture of the *IgH* locus. At the architectural level, CSR requires spatial juxtaposition (synapsis) of the donor Sμ and acceptor S regions, achieved through cohesin-driven loop extrusion that brings the 5′Eμ enhancer into proximity with the 3′ regulatory region (3′RR), a process that creates a ‘CSR centre’ (CSRC) where AID-initiated DSBs are brought into proximity for deletional recombination (69–71). We propose that the RNA hub described by Mikhova et al. (40) may represent the biochemical microenvironment of this CSRC, integrating LLPS-driven factor concentration with loop extrusion-mediated topology. Meanwhile, 53BP1-RIF1 assemblies stabilize these configurations and restrict DNA damage signaling to appropriate topological domains, thereby enforcing productive recombination while suppressing aberrant rearrangements (72). Enhancer-derived RNAs further contribute by engaging the hnRNPL–CstF64 complex to scaffold 53BP1 and Ku80 recruitment at both switch and enhancer regions (41). Efficient assembly of these structures requires chromatin preconditioning by the segregase VCP/p97, which removes repressors like L3MBTL1 to enable proper 53BP1 condensate formation at S regions (73). (**Figure 6**). Of note, not all chromatin structures discussed here necessarily arise through canonical LLPS. Some examples derive from non-B-cell systems and are therefore discussed as mechanistic analogies rather than direct CSR evidence, highlighting shared organizing principles based on multivalency, cooperativity, and dynamic compartmentalization.

Together, these findings support a model in which LLPS-associated assemblies function as integrative organizers of chromatin dynamics, bridging nanoscale repair factor clustering with mesoscale genome topology. In CSR, this multiscale organization is essential for coordinating chromatin remodeling, repair pathway choice, and long-range synapsis to ensure faithful antibody diversification while preserving genome integrity.

## 3 Genomic consequences: dysregulated LLPS in disease

The precise spatiotemporal regulation afforded by LLPS is not merely facilitative but essential for preserving genome integrity. While the preceding sections outlined how physiological condensates organize transcription, DNA damage signaling, and repair during CSR, the intrinsically dynamic and metastable nature of these assemblies renders them particularly susceptible to perturbation. Dysregulation of LLPS—manifesting either as impaired condensation, in which functional repair hubs fail to assemble or remain insufficiently stable, or as hyper-stable pathological condensation, in which assemblies become rigid, mislocalized, or resistant to dissolution—emerges as a direct driver of defective CSR, genomic instability, and human disease.

Impaired condensation disrupts the formation of essential repair microenvironments, resulting in loss of function. In CSR, perturbation of 53BP1- or HNRNPU-dependent condensates compromises the recruitment and spatial coordination of NHEJ factors, leading to inefficient recombination and increased chromosomal instability (15) (**Figure 4**). In parallel, long-range synapsis of donor and acceptor S regions relies on 53BP1-driven chromatin compaction; disruption of associated architectural regulators such as NIPBL alters *IgH* locus topology and undermines productive repair. Notably, these defects mirror features observed in Cornelia de Lange syndrome, underscoring the broader relevance of LLPS-dependent genome organization to human developmental disease (54).

Beyond CSR, loss of physiological LLPS function in tumor suppressors contributes to oncogenesis. The speckle-type BTB/POZ protein SPOP normally forms tumor-suppressive liquid-like droplets that inhibit protein aggregation; cancer-associated mutations disrupt this LLPS capacity, compromising its tumor suppressor role (74, 75). Similarly, truncating mutations in the histone demethylase UTX eliminate its scaffolding function as an LLPS hub for chromatin modifiers, thereby promoting tumorigenesis (76, 77). These examples highlight how failure to assemble appropriate condensates phenocopies genetic loss of function at the level of nuclear organization.

Conversely, hyper-stable or aberrant condensates promote misallocation of repair machinery and gain of pathological function. In CSR, hyper-stable condensation of AIDΔC mutants sequesters functional AID, providing the direct molecular mechanism for CSR failure in Hyper-IgM syndrome (16, 78). This dominant-negative trapping of AID^WT illustrates how excessive or improperly regulated condensation can phenocopy loss of activity, despite preserved catalytic potential (**Figure 3**). Beyond B cells, dysregulated phase separation drives oncogenesis through acquired LLPS capacity. Oncogenic fusion proteins like NUP98-HOXA9 and EWS-FLI1 gain LLPS capability through intrinsically disordered regions, driving aberrant chromatin looping and super-enhancer formation that activates pro-tumorigenic gene expression programs (79). Notably, mutant forms incapable of LLPS lose their tumorigenic potential, establishing phase separation as a critical determinant of oncogenicity. Similarly, persistent signaling condensates formed by mutated SHP2 drive unrestrained MAPK activation, illustrating how solidified condensates can lock signaling pathways in an oncogenic state (80).

This continuum of dysregulation—from loss of essential physiological condensates to gain of pathological phase separation—highlights that maintaining precise condensate dynamics is a decisive determinant of genome integrity. In B cells, disruption of this balance manifests as defective CSR, immunodeficiency, and lymphomagenesis. More broadly, these principles establish LLPS as a unifying mechanistic framework linking nuclear organization, genome maintenance, and disease, and point toward therapeutic strategies aimed at restoring appropriate condensate dynamics rather than simply targeting individual molecular components.

## 4 Future perspectives and concluding synthesis

### 4.1 Outstanding questions and methodological frontiers

Despite the compelling model of the LLPS-driven switchosome, several fundamental questions remain. A primary challenge is bridging the gap between *in vitro* observations and *in vivo* functionality. How can we definitively prove the existence and necessity of a liquid-like condensate at the native *IgH* locus? Addressing this question will require experimental strategies that move beyond global perturbations toward locus-specific manipulation of condensate assembly. Emerging approaches combining CRISPR-based genomic targeting with optogenetic or multivalent scaffold systems (e.g., optoDroplet-derived platforms, Corelets, or related tools) may enable controlled nucleation or dissolution of condensate-like structures at defined chromosomal coordinates, allowing direct functional testing of their contribution to CSR. Such approaches may help bridge the current gap between artificial condensate induction and precise manipulation of condensate assembly at endogenous genomic loci, thereby enabling more rigorous tests of causality. Complementary advances in live-cell super-resolution imaging and *in situ* structural approaches will further enable visualization of condensate dynamics, composition, and spatial organization within the nuclear environment (81–83). Another major challenge lies in establishing causality by distinguishing the effects of protein abundance from the material properties of the assembly itself. Mutational strategies that selectively perturb intrinsically disordered regions or multivalent interaction interfaces—while preserving catalytic domains—may help determine whether condensate formation per se is required for efficient repair pathway coordination. At the same time, the specificity paradox demands explanation: in a nucleus enriched with IDR-containing proteins and RNA, what molecular features ensure that condensate assembly occurs selectively at immunoglobulin switch regions? A plausible answer may lie in a unique molecular grammar composed of AID interaction surfaces, transcription elongation factors such as Spt5, G4-RNA scaffolds, and local chromatin modifications that collectively nucleate higher-order assemblies at S regions. Finally, the mechanisms regulating the material state of these assemblies remain poorly understood. The transition of AID from a soluble state to pathological solid-like aggregates in AIDΔC mutants suggests that precise control over condensate viscosity is critical for proper CSR function. Future studies integrating quantitative live-cell imaging with single-cell genomic and transcriptomic approaches may help bridge the “timescale paradox, “ whereby rapid molecular dynamics occurring on the order of seconds ultimately influence long-term immunological outcomes such as antibody diversification and immune responses. Investigating how post-translational modifications—including phosphorylation and PARylation of factors such as 53BP1 and HNRNPU—modulate condensate properties may therefore provide key insights into the regulatory logic governing CSR repair environments. Ultimately, deciphering this regulation may open new therapeutic avenues. Rather than relying on nonspecific disruptors of phase separation, future strategies may aim to selectively modulate pathological condensate states while preserving physiological nuclear organization, offering potential interventions for cancers and immunodeficiencies linked to dysregulated CSR (84). Importantly, future studies must also distinguish LLPS-dependent mechanisms from alternative processes that can generate similar spatial organization, including chromatin looping, molecular crowding, and polymer-based scaffolding. Together, these emerging experimental and conceptual approaches will be essential for establishing whether biomolecular condensation represents a causal organizing principle of CSR or instead reflects a higher-order manifestation of underlying chromatin and transcriptional dynamics.

### 4.2 LLPS as a unifying principle in B cell biology

The LLPS paradigm, while firmly emerging in CSR, likely extends to other AID-mediated processes. It is a compelling hypothesis that somatic hypermutation (SHM), which requires processive AID activity on the same DNA strand, is governed by a distinct, more processive condensate state. We propose that divergent physicochemical properties of the AID machinery—such as differences in client protein recruitment, condensate stability, or material state—could dictate the binary decision between processive mutagenesis in SHM and deletional recombination in CSR. Moreover, this framework provides a new lens through which to view primary immunodeficiencies. Beyond known AID mutations, it is plausible that a class of “LLPS-opathies” exists—disorders caused by hypomorphic mutations in 53BP1, HNRNPU, or other complex members that do not ablate protein expression but subtly disrupt the multivalent interactions necessary for productive condensate assembly, leading to a primary antibody deficiency.

### 4.3 Concluding synthesis

In summary, the evidence reviewed here supports LLPS not as a passive organizing phenomenon, but as an active and indispensable regulatory principle that resolves the spatiotemporal constraints inherent to CSR. This framework delineates a continuous physicochemical pathway spanning PARP1-mediated sensing of transcription-associated stress, RNA-guided recruitment of AID, assembly of the 53BP1–HNRNPU–dependent “switchosome” that enforces pathway choice favoring C-NHEJ over A-EJ, and large-scale reorganization of *IgH* chromatin topology to enable productive synapsis. By integrating these steps, an LLPS-centered model reframes CSR from a series of probabilistic molecular encounters into a coordinated, compartmentalized cascade governed by emergent material properties. The fidelity of this process is fundamental to adaptive immunity, whereas its disruption—whether through failure to assemble essential condensates or through pathological stabilization of aberrant assemblies—provides a direct mechanistic route to genomic instability, immunodeficiency, and lymphomagenesis. Ultimately, elucidating the molecular logic and biophysical properties of CSR-associated condensates offers a unifying framework for understanding genome maintenance in B cells and suggests that therapeutic modulation of condensate dynamics may represent a complementary strategy for controlling immune responses and targeting B cell malignancies.
